# Medical and Physical Intelligence

**Published:** 1820-10

**Authors:** 


### Medical and Physical Intelligence.

### TRANSACTIONS OF SCIENTIFIC SOCIETIES.

ROYAL *Academy of Sciences of Paris.*—June 19. M. DUMERIL gave an account of a Memoir, by M. JULIUS CLOQUET, on the *Lacrymal Canals of Serpents.* It has been said in all times, since it was stated in so express a manner by ARISTOTLE, that serpents have no eye-lids; that the surface of their eyes is always dry; and all authors have asserted that those animals produce no tears. M. Cloquet combats these statements, by the results of a series of careful anatomical researches. He shows that serpents have a considerable quantity of lacrymal fluid in front of the eye, which organ is movable under a fixed and transparent eye-lid; and that the presence of tears and of the conjunctiva, which has the form of a sac, must facilitate this motion.

The conclusion of a Memoir on *Yellow-Fever*, by M. MOREAU DE JONES, was read. He furnishes a list of the eruptions of what he supposes to have been this disease, during the last 325 years; of which he enumerates 191: 101 in the torrid, and the rest in the temperate zone.

In a late sitting of the *Society of the Faculty of Paris*, Baron PERCY presented an account, which he received from M. MAGUIN, surgeon to the Chasseurs de la Meuse, of a portion of the right ear of a soldier having been entirely separated from the rest, and which had perfectly adhered and grown to it, on being re-applied by the surgeon just named. The Faculty entertained some doubt about this as a fact. M. LAURENT is said, by M. DE LENS (in the *Bibliotheque Medicale*), to have witnessed a similar circumstance, which is not less probable than that of the re-union of a part of a finger, of which Dr. BALFOUR of Edinburgh has so lately published an example; and the other curious things of this kind, of which GRAEFE has given a full history in his *Rhinoplastik.*

THE question proposed by the *Cercle Medical of Paris*, in 1819, not having been treated in a satisfactory manner, is again proposed for the next year: it is "To determine the influence of pathological anatomy on the progress of medicine in general, and especially on the diagnosis and treatment of internal diseases."

The Society requests the concurrents, "1°, to enquire whether or not pathological anatomy may, in its present state, give rise to applications and interpretations injurious to science; 2°, to indicate the means which they believe to be the most proper to prevent these inconveniences: in a word, it engages them to take the sense of the word *influence* in its bad, as well as in its good, relations."

The prize will be a medal of the value of three hundred francs. The memoirs, written in French or Latin, are to be sent, with the usual forms, to M. le Docteur Chardel, Secrétaire général du Cercle Médical, Rue Cassette, No. 26, before the 1st of July, 1821.

---

A patient, forty-one years of age, after having been in several hospitals, entered the Hôtel-Dieu, to be treated for several fistulas, which he had had for several years, in the perineum. M. Dupuytren, having passed a probe into the fistulas, encountered several bodies, which he found to be stones. The patient was then sounded, and the stones were again felt, not in the cavity of the bladder, but in the prostate gland. Several incisions were made in this part, in different directions; twelve stones, with smooth articulated surfaces, were extracted; the patient experienced no untoward accidents, and left the hospital perfectly cured.

The stones, on being analyzed by Thenard, were found to be composed of 86 parts of phosphate of lime, 13 of animal matter, and some traces of carbonate of lime.—*Journal Universel des Sc. Medic.* August 1820.

---

We have received two letters respecting the *remedy for Tænia*, noticed in the last Number of this Journal: one from Mr. Lang, of Kensington, who suggests "that it is the fruit of the *cucurbita citrullus* which is meant; as I (Mr. L.) remember to have heard it once recommended for some verminous disorder." Our other correspondent, G. L., believes it to be the same plant; and refers, in proof, to "vol. ii. p. 382, of *Histoire abregée des Plantes usuelles*, par Chomel, augmentée par Mallard, Paris 1804." M. Virey, in his *Histoire Naturelle des Medicamens*, says, the *citrouille* is the *cucurbita pepo* (common *pumpkin* or *gourd*), not the *C. citrullus*. The *cucurb. cit.* occurred to us; but we did not suppose that any part of this plant, the *water-melon*, could possess the powers indicated by M. Mongeny. It is eaten freely by the lower class of people in Provence, cooked in sweet wine, as the common pumpkin is amongst us, and does not appear to differ remarkably from it in its qualities. However, Mr. Lang's remark makes it probable that this plant was really signified.

---

*School of Physic in Ireland.*—The Session commences the first Monday in November. Courses of Lectures, &c.: Anatomy, Physiology, and Surgery, including Diseases of the Skin, 4 guineas; Pathology, 1 do.; Chemistry, 4 do.; Botany (in the summer), 4 do.; Institutes of Medicine, 4 do.; Materia Medica, 4 do.; Practice of Medicine, 4 do.; Clinical Lectures, 3 do.; Diseases of the Eye, 1 do.; Toxicology and Medical Jurisprudence, 2 do.; Midwifery, 2 do.; Practice of the Hospital, during one year, 3 do.; Anatomical Demonstrations, and dead subjects provided, 6 do.; the same, a second season, 4 do.; Instructions in Operative Surgery, and dead subjects, 5 do.; Instructions in Operative Chemistry, 6 do.; Botanical Demonstrations, 1 do.; Practical Pharmacy, 2 do.

*Obs.* Two kinds of medical degrees are granted in the University of Dublin: 1st, those of Bachelor and Doctor of Physic, which rank with the same in Oxford and Cambridge; 2d, the *Testimonium*, which corresponds with the medical degree of Edinburgh and Glasgow.

---

*Animal Magnetism.*—The following proposal has been made and published, (in the *Archiv für den thierischen Magnetismus*,) by M. Chastenet de Puysegur, the first animal magnetiser in all France. "Let me be invited, let me even be ordered, if it be wished, to confine myself for six weeks in an hospital for the deaf-and-dumb, that I may lay in wait for, and seize, the occasion of transient disease in some of the pupils, to magnetize them. Let them not be informed in any way of what they are to experience; but let them suppose that I am present as an ordinary physician. Several of them, unavoidably, will become somnambulists, and in the number there will certainly be found some who are more mobile than the rest, who will become sensible of the influence of my *animal* magnet. Well then, if in this state I do not make them act, walk, take what I choose, or answer by writing all the questions I shall put to them relative to their welfare and their health, not from my speaking to them, since they would not hear me, but solely by the mental impulse of my will; let it be decided that there is neither magnet nor magnetism in man. I will even subscribe to the decision that, during the more than thirty years which I have affirmed and published its existence, I have been only an apostle of error, of illusion, and of deceit."

When a sect of fanatics, about a century since, pretended to have the power of raising up to life a dead body, and proposed a test of their ability, the government acceded to their request. When we remember that there has been appointed a professor of animal magnetism at the Royal Academy of Sciences at Berlin, (Dr. Wolfert;) that this professor superintends an infirmary for its application; and that Russian and Austrian physicians are appointed by their governments to attend him, and learn his method, we must acknowledge that the subject has assumed an air of sufficient importance to induce some government to consent that M. Chastenet de Puysegur shall have the opportunities he desires.

---

*On the Vegetation of Bulbous Roots in Water.* By Mr. Murray.—I made last winter some experiments on bulbs growing in water, chiefly with reference to a transition into varied media; and a short detail of some of them here, while they afford information with regard to the functions of the roots, may not be deemed uninteresting. The bulbs were those of the hyacinth.

The Dutch keep the hyacinth, when newly placed on the bulb-glass, for some time in the dark, to induce good roots; and for this purpose, by way of comparison with other bulb-glasses, I used coloured ones. The fibres of the root seem generally to attain a greater longitude in coloured glasses, in the green especially. In the blue, the fibres are marked by the singularly strong ones which subsequently surround the margin.

The very strong swollen fibres which characterize the latter stage of the plant, is perhaps connected with the evolution of the flower at that period when it should declare itself. At any rate there are two distinct series of roots, and the primitive more minute fibres shrink when the others emerge.

A bulb fed with a solution of carbonate of ammonia, continued healthy, and flowered well; but the roots did not greatly elongate.

One hyacinth-bulb was inured to repeated additions of salt-water, 'till sea-water was finally used: it continued to grow, but was not so luxuriant as the others.

A bulb fed with diluted pyroligneous acid, did not seem materially affected by the new medium, and the fibres seemed to have decomposed the acid matter in contact.

Repeated renewals of the water seem to insure health and luxuriance, and more rapid growth. The probability is, that there is an appropriation of the air contained diffused through the water, which is in time exhausted; and, according to M. Gay-Lussac, the air contained in water contains excess of oxygen.—*Annals of Philosophy*, No. xciii.

---

*Agave Americanum.* By Mr. MURRAY.—THE *agave Americanum* is said to flower only once in a century. This much we know at any rate, its flower is a rare phenomenon in the British isles. Not a few, however, may be seen in flower on the sides of the high-way from Terracina to Capua, on the road to Naples. I think it obvious, from an attentive examination of many of these plants, that this agave flowers only *once;* and that, having provided for its perpetuity by an offset, the whole energies of the plant are expended in the flower, which being matured, it perishes. The most magnificent flower of this species I ever saw, was growing on an exposed rock in one of the Borromean islands, called Isola Madre, in the Lagomaggiore, north of Italy. I measured the flower-stem, or pedunculus, and found it twenty-eight and half feet in altitude; and its circumference, where it emerged from the leaves, two feet ten inches: it was a superb spectacle.

I was inclined to consider this plant as indigenous to Italy; but Sir James E. Smith tells me he is not of this opinion. It is, however, common in that country. It crowns the walls of the city of Genoa, where it even flowers; and I have seen whole fields near Ponterculc fenced-in entirely with the agave Americanum.

The plant sketched in *fresco* on one of the walls of Pompeii, supposed to be an agave, and which would assign a distant date to its introduction into Italy, does not appear to me, (and Dr. J. F. Schouw joins in the opinion,) ever to have been intended for the plant in question.—*Ann. Phil.*

*Crystallization of Platina.* By G. B. Sowerby, Esq.—I am not aware that any thing decided is known respecting the crystallization of this substance. Bournon, it is true, has mentioned some grains of platina which have a distinct form ; but these appear to have been a deposition of platina upon some other substance which has afterwards been decomposed and gone, for they are hollow and mammillated upon their outer surfaces. I have some of these, and I have reason to think that they have been formed over palladium, for some externally mammillated portions of platina which accompany them are still adherent to native palladium. But, in looking over a small parcel of platina very lately, I discovered several pieces which had a perfectly lamellar structure, and a distinct cleavage; and one of them which showed the four faces forming the solid angle of an octahedron. I have preserved this little fragment, as being perfectly demonstrative for the inspection of any who may think the subject sufficiently interesting.—*Ann. Phil.*

### MONTHLY CATALOGUE OF MEDICAL BOOKS.

### REPORT OF DISEASES.

THE past month has been unusually salutary: but few cases of cholera morbus, the ordinary prevalent malady in a manner proper to the season, have occurred. Rheumatic affections have, however, still continued to present themselves very abundantly; and pulmonary inflammation, of different kinds, has been remarkably common, especially amongst the lower orders of people, amongst whom, also, hemoptysis has been observed almost as frequently as in the spring season. The rapid and great alternations of temperature, and the cold winds which have been experienced, will explain this unusual character of the prevalent diseases of the month of September. Disorders of the abdominal viscera have been proportionately rare, if we except in children, with whom, we think, the reverse has been the case. Many instances of signs of severe cerebral irritation, apparently dependant on intestinal disorder, as noticed in our last Report, have continued to occur to our observation. In one fatal case of this kind, there were found after death the ordinary signs of recent inflammation of the arachnoid membrane in the cranium, and numerous spots of ulceration, evidently of long standing, dispersed in the mucous membrane of the small intestines, with the appearances of only very recent inflammation of the same membrane, throughout almost the whole of the duodenum and the ileum. Yet the mother of this child, which was five years old, said that the child's bowels had not been disordered, until about a fortnight before its death, for many months previously. It is very common to find those of the lower orders assert this, though the clearest evidences of very severe and long-continued disease is discovered on dissection. The truth seems to be, that they know nothing about the matter, and assert positively what they have only negative information of. It is a fearful thing to contemplate the ravages of intestinal disease amongst the poor children of this metropolis. Their diet is probably worse than that of any other children in Great Britain, excepting, perhaps, those of our large manufacturing towns. A child will thrive very well on potatoes and milk, or on oatmeal, alone; but it cannot be supposed that any thing else than disorder can result from the use of such a pernicious and irregular diet as the generality of the children alluded to are submitted to.

We hear from all quarters favourable reports of the colchicum in the diseases stated in the last Report, and in London especially it is coming into very general use. The remark is adventitious to the subject of this Report; but it is important that the profession should know that the wine of it, prepared by different chemists, varies much in regard to strength: some of them, indeed, prepare it only from the seeds, and give this when *vin. colchici* is prescribed. We prefer the preparation from the bulbs, and always employ it: perhaps we have not given the seeds a fair trial, but, as far as our observation has gone, we are led to think the latter a comparatively inefficacious medicine.

